# Evaluation of the frequency and severity of seasonal allergic rhinoconjunctivitis caused by grass pollen in children with hazelnut sensitization

**DOI:** 10.1186/2045-7022-3-S3-P76

**Published:** 2013-07-25

**Authors:** O Cavkaytar, B Buyuktiryaki, I Kizilpinar, E Arik Yilmaz, A Tuncer, C Sackesen

**Affiliations:** 1 Department of Pediatric Allergy and Asthma, Hacettepe University Medical Faculty, Ankara, Turkey

## Background

The high frequency of pollen sensitization has been reported among peanut and tree nuts sensitized patients in our previous study. The antigen homology of peanut and tree nuts antigens with betula was previously shown. However there is a lack of knowledge about the cross-reactivity between hazelnut and grass pollen antigens. The aim of this study is to find out if there is a difference in the frequency and the severity of allergic rhinoconjunctivitis (ARC) in grass pollen sensitized children when hazelnut sensitization is coupled.

## Methods

Seventy children diagnosed with allergic rhinitis, food allergy or egzema are divided into three groups as “only grass pollen sensitized”, “only hazelnut sensitized” or “both pollen and hazelnut sensitized”. Double-blind-placebo-controlled food challenge tests determined children who are allergic or tolerant to hazelnut. Symptoms and medications for ARC and asthma were scored from 0 to 3 during the season of grass pollen from 1^st^April to 31^st^July 2012. Symptom and medication scores for ARC and asthma were analyzed by ''Area Under Curve'' (AUC) which was calculated by NCSS (Number Cruncher Statistical System), ''p for trend'' and Kruskal-Wallis tests.

## Results

ARC symptom scores (ARCSS) and medication scores, were lower in “both hazelnut and pollen sensitized” group compared to “only pollen sensitized” group (p<0.05). There was a tendency for decrement of ARCSS among four groups; “only pollen sensitized” (162, 99-526) (median, interquartile range), “both pollen and hazelnut sensitized but tolerant to hazelnut” (162, 33-286), “both pollen and hazelnut sensitized but allergic to hazelnut” (104, 60-182) and “only hazelnut sensitized” (34, 2-89) (p<0.0001, p for trend). Grass pollen IgE was higher in “only pollen sensitized” group (42, 8-77 kU/L) than “both pollen and hazelnut sensitized but tolerant to hazelnut” (20, 4-149), “both pollen and hazelnut sensitized but allergic to hazelnut” (9.2, 0.7-95.8) and “only hazelnut sensitized” groups (0.1, 0.1-0.5) (p<0.0001).

## Conclusion

Grass pollen sensitization is common among hazelnut sensitized children. Our results denote that the severity of seasonal ARC may change according to the presence of hazelnut sensitization and its clinical reactivity. Symptom severity of ARC and grass pollen spesific IgE levels are lower in both hazelnut and grass pollen sensitized children than that of only pollen sensitized children.

## Disclosure of interest

None declared.

